# Phytochemical and antioxidant screening of *Moringa oleifera* for its utilization in the management of hepatic injury

**DOI:** 10.3389/fnut.2022.1078896

**Published:** 2022-12-15

**Authors:** Noor Younis, Muhammad Issa Khan, Tahir Zahoor, Muhammad Naeem Faisal

**Affiliations:** ^1^National Institute of Food Science and Technology, University of Agriculture, Faisalabad, Pakistan; ^2^Institute of Physiology and Pharmacology, University of Agriculture, Faisalabad, Pakistan

**Keywords:** *Moringa oleifera*, acetaminophen, hepatic injury, antioxidant potential, silymarin

## Abstract

**Introduction:**

Phytochemicals present in *Moringa oleifera* (*M. oleifera*) leaves have performed several physiological functions in human system such as anticarcinogenic, antidiabetic, antioxidant, immunomodulatory, hepatoprotective and antiatherogenic functions.

**Methods:**

Phytochemical and antioxidant potential of *M. oleifera* leaves extracts were measured. Histopathology, biochemical analysis, and gene expression tests were performed on serum, blood, and liver in animal model.

**Results and discussions:**

The toxic dose of N-acetyl-para-aminophenol (APAP) induced severe structural and functional changes in liver. Pre-treatment with *M. oleifera* ameliorated organ injury by normalizing the level of liver biomarkers and serum proteins. A low expression level of MAPK-8, TRAF-4, and TRAF-6 genes was observed in the *M. oleifera* treated group in comparison to positive control (hepatotoxic rats). *M. oleifera* leaves pretreatment amended APAP induced apoptosis and replenished hepatic cells. *M. oleifera* leaves extract as low-cost and sustainable treatment could be used in pharmaceutical industry for reducing hepatic degenerative changes in non-communicable diseases.

## 1 Introduction

*Moringa oleifera (M. oleifera)* is one of the most promising medicinal plants (1) among all plant species and belongs to the *Moringaceae* family. All parts of *M. oleifera* are valuable including pods, flowers, leaves, and seeds (2). *M. oleifera* has shown its therapeutic potential against several human pathologies; dyslipidemia, hypertension, asthma, diabetes, infection, rheumatoid arthritis, cancer (3), and wound healing (4). A lot of *in vivo* and *in vitro* models have shown the worth of using *M. oleifera* for anti-inflammatory, anti-diabetic, antibacterial, antioxidant, anti-microbial (5) and anti-fibrotic properties (6). *M. oleifera* plant parts were analyzed for their anti-nutritional metabolites, which showed the dominance of flavonoids, kaempferol, quercetin, vanillin, and glucosinolates (7). Flavonoids are major free radical scavengers, thus minimize the risk of illnesses.

Liver pathologies account for about 2 million deaths every year worldwide. Cirrhosis stands at the top among 20 non-communicable diseases that causes disability-adjusted life years and life lost years, causing mortality of 1.6 and 2.1% (8). Globally, hepatic disorders account for increased mortality and morbidity and their mitigation is the biggest challenge to the society (9). Prescribed medications such as aspirin, naproxen, ibuprofen, diclofenac, and acetaminophen account for 50% of liver failure cases (10). Acetaminophen (APAP) is a widely consumed antipyretic and analgesic drug worldwide. Unfortunately, over-consumption of acetaminophen is associated with acute liver failure and drug-induced liver diseases in animal and human models (11). Saturation of metabolic pathways occurs at toxic doses that results in increased synthesis of toxic metabolite, *N*-acetyl-*p*-ben zoquinoneimine (NAPQI), and decreased antioxidant activity in the body (12). Evidence is there regarding lipid peroxidation, mitochondrial dysfunction, and reactive oxygen species synthesis about acetaminophen-induced hepatotoxicity (13). Dietary flavonoids, having the ability to prevent oxidative stress, chromosome instability linked to diseases and genetic mutation, is gaining much attention worldwide (14). *M. oleifera*, a plant with potent anti-inflammatory properties, has also been reported to possess hepato-protective action against inter-tubular drugs (15). In developing countries, *M. oleifera* is an extensively used vegetable because of its diverse nutrient range. The presence of secondary metabolites in *M. oleifera* makes it even more well-intentioned. Quercetin, apigenin, kaempferol, and isorhamnetin are flavonoids abundantly present in *M. oleifera* (16). Quercetin, present in the *M. oleifera*, is known to be an effective hepatoprotective agent (17). Quercetin belongs to the family of flavones, having a strong potential to prevent or delay oxidative degradation in the body (18). Hydroxyl group present in quercetin act as an electron donor and helps to scavenge free radical (19). The chemical structure of quercetin is also capable to suppress the expression of low-density lipoprotein receptors (LDLR) and enhance the uptake of low-density lipoprotein (LDL) toward a liver cell (20). Compounds with the potential to minimize the effect of reactive pro-oxidant species are useful to prevent hepatic injury induced by specific byproducts and *M. oleifera* is assumed to serve protective action against hepatopathy (21). Keeping in view the above-mentioned facts, the present study is designed to assess the phytochemical potential of *M. oleifera* against acetaminophen-induced oxidative stress and its modulatory effects on liver biomarkers, liver histology, and MAPK downstream JNK Pathway. This study would enlighten the knowledge on the use of *M. oleifera* leaves and its bioactive constituents in the field of clinical research.

## 2 Materials and methods

### 2.1 Plant material

Leaves of the *M. oleifera* plant were obtained from the Department of Crop Physiology, University of Agriculture, Faisalabad, Pakistan. The obtained samples were cleaned, sun-dried, and ground to a fine powder for phytochemical analysis.

### 2.2 Preparation of *Moringa oleifera* leave extract

Extract of *M. oleifera* leaves was prepared by making slight modifications to the method used by Vats and Gupta (22). For this purpose, 10 g *M. oleifera* leave powder was extracted with 100 ml (75%) ethanol and placed on an orbital shaker (KS-260 Edmund Buhler mg H-Ks 15, Germany) for 3 h at 280 rpm. Samples were then centrifuged (MPW^®^-352R, Med. Instruments) at 5,000 rpm for 15 min. The supernatant of the samples was again filtered with Whatman filter paper, and the filtrates obtained were carried to a rotary evaporator for evaporation of alcohol (ethanol residue ∼3–5 μl/ml) under reduced pressure at 50°C, 120 rpm. The extracts obtained were stored at –18°C until further analysis.

### 2.3 Physicochemical screening

#### 2.3.1 Total phenolic content

**FIGURE 1:**
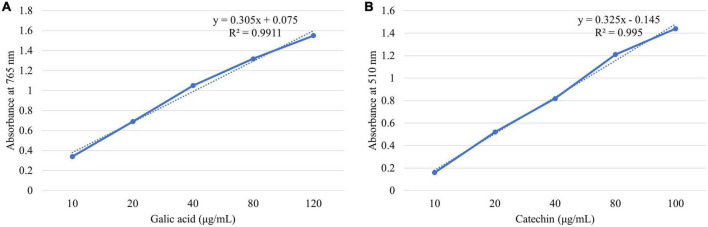
Calibration curve of **(A)** gallic acid and **(B)** catechin.

Total phenol in *M. oleifera* leaves was measured through the Folin-Ciocalteau assay (FCA) with slight modifications to the method described by Wu et al. (23). For this purpose, 50 μl of each extract was taken in a volumetric flask with added FCA reagent 250 μl and 20% w/v sodium carbonate 500 μl (freshly prepared). The mixture was vortexed initially and then kept at room temperature for 30 min for incubation purposes. The absorbance was checked at 765 nm *via* a UV–Vis spectrophotometer (T80, PG 95 Instruments, United Kingdom). Gallic acid calibration curve (Figure 1) was developed by making standard solutions of 10–120 μg/ml concentration (*y* = 0.305*x* + 0.075, *R*^2^ = 0.9911). The total phenols present in *M. oleifera* were expressed as mg gallic acid equivalent (GAE) per g of *M. oleifera* leave extract using a calibration curve.

#### 2.3.2 Total flavonoid content

Total flavonoid from *M. oleifera* was determined by following the method of Wu et al. (23) with slight modifications. Each sample extract of 1 ml is diluted with 4 ml distilled water (dH_2_O). Concurrently, add 0.3 ml freshly prepared NaNO_2_ (5%, w/v) into the flask. The mixture was left for 5 min and the 0.3 ml of freshly prepared AlCl_3_ (10%, w/v) was added. After leaving the mixture again for 6 min, 2 ml of NaOH (1 M) and 2.4 ml of dH_2_O were added to the flask and were mixed thoroughly. The absorbance was checked at 510 nm *via* UV–Vis spectrophotometer (T80, PG 95 Instruments, United Kingdom) against a blank prepared with water. A catechin calibration curve (Figure 1) was obtained at different concentrations (10–100 μg/ml, *y* = 0.325*x* - 0.145, *R*^2^ = 0.995). Total flavonoids in *M. oleifera* were calculated by comparing with the calibration curve of catechin and were expressed as mg catechin equivalent (CTE) per g of *M. oleifera* leave extract.

### 2.4 Antioxidant assays

#### 2.4.1 2,2-diphenyl-1-picrylhydrazyl radical (DPPH•)

The ability of the *M. oleifera* to the donation of a hydrogen atom or an electron was assessed following the protocol of Xie et al. (24) by using a methanolic solution of DPPH•. For this purpose, 2.5 ml DPPH• solution (0.5 mM) was mixed with 0.5 ml of *M. oleifera* extract. The mixture was vortexed and incubated at room temperature for 30 min in a dark place. Afterward, absorbance was checked at 517 nm *via* a UV–Vis spectrophotometer (T80, PG 95 Instruments, United Kingdom) against a blank. Results were expressed as percent inhibition of DPPH radical by using the following equation:


%DPPH•

$$=\frac{\mathrm{Absorbance}\,\mathrm{of}\,\mathrm{control}-\mathrm{Absorbance}\,\mathrm{of}\,\mathrm{sample}}{\mathrm{Absorbance}\,\mathrm{of}\,\mathrm{control}}\times 100$$

#### 2.4.2 The ferric reducing ability of plasma

The ferric reducing antioxidant potential of *M. oleifera* extracts was determined according to the protocol of Brito et al. (25) with slight modifications. Accordingly, FRAP working solution was made by mixing acetate buffer 25 ml (pH 3.6, 0.3 M), with 2,4,6-tris-(2-pyridyl)-S-triazine 2.5 ml (10 mM) and ferric chloride 2.5 ml (20 mM). Then 280 μl FRAP solution was added to 20 μl of each extract and kept for 30 min in a dark environment for incubation at room temperature. The absorbance was checked at 593 nm *via* UV–Visible spectrophotometer (T80, PG 95 Instruments, UK). A calibration curve was prepared by using FeSO_4_ and the results were expressed as μM FeSO_4_/g of MO.

### 2.5 Animal groupings and drug administration protocol

Bio-efficacy trial was performed to evaluate the preventive action of *M. oleifera* leaves against drug-induced hepatic injury. To achieve this goal, 40 Wistar rats (200–250 g) at the 12th week of age were kept in the animal house of the National Institute of Food Science and Technology, University of Agriculture, Faisalabad, Pakistan. The rats were provided with standard diet and water *ad libitum* and kept at constant room temperature (24 ± 2°C) under a 12 h light/dark cycle for 1 week before the start of the experiment, so they can get acclimatized to the environment.

Rats were divided into four groups (Table 1) each having 10 animals. All the experimental subjects were marked on the tail carefully with different colors to identify each animal group and its specified allotted group. A preliminary study, based on the previous findings (26–28), was conducted to select the effective dose (200 mg/Kg BW) of *M. oleifera* leaves. After 1 week of acclimatization, pretreatment of Silymarin (200 mg/kg of BW) and *M. oleifera* (leaves) powder (200 mg/kg of BW) was given to Group 3 and Group 4, respectively from the day 7th to the day 21st of the study. Group 1 and Group 2 received a normal diet throughout the study. On the 21st day, all groups except Group 1 received a high dose of acetaminophen (APAP) (200 mg/kg (29) of BW) by dissolving in warm saline. After APAP administration rats were fasted for 8 h to induce acute liver toxicity and then sacrificed to collect blood samples, the liver was collected and stored abruptly for histopathology and gene expression analysis.

**TABLE 1:** Treatment plan to observe the hepatoprotective effect of *Moringa oleifera* in APAP induced hepatotoxic rats.

Groups	Treatments
G_0_: Negative control	Normal diet
G_1_: Positive control	Normal diet + APAP (200 mg/kg of BW)
G_2_: Standard drug	Normal diet + Silymarin (200 mg/kg of BW) + APAP (200 mg/kg of BW)
G_3_: Treatment	Normal diet + *M. oleifera* (200 mg/kg of BW) + APAP (200 mg/kg of BW)

### 2.6 Biochemical analysis

Rats were weighed at the end of the study before decapitation. Blood was drawn after decapitation in purple-cap vials, which were coated with ethylenediamine tetraacetic acid (EDTA) for hematological analysis and yellow-cap vials, non-coated tubes, which were centrifuged for 15 min at 15,000 rpm at 4°C to separate serum for analyzing serum specific biomarkers. The organ of interest (liver) was separated and immediately stored in a 10% formalin solution for histological examination. Biochemical parameters include alanine transaminase (ALT), alkaline phosphatase (ALP), aspartate transaminase (AST), Serum bilirubin, Gamma-glutamyl transferase (GGT), albumin, globulin, and A/G ratio, which were analyzed by using an automated serum analyzer (Bio-Ray 310 diagnostic) and biochemical kits of Merck (private) limited, Pakistan. The oxidative stress parameters were assessed by measuring serum levels of total oxidant status (TOS) and total antioxidant capacity (TAC) by using a microplate spectrophotometer (Thermo Scientific Multiskan GO™ with SkanIt software 4.1) according to the protocol described by Hussain et al. (30).

### 2.7 Histopathological study of liver

The liver was kept in 40% formalin solution for histopathological examination immediately after decapitation of animals. The tissue obtained from each organ was carved into 5 μm thick sections with the help of a microtome and mounted on glass slides. Staining was done with periodic acid-Schiff and hematoxylin-eosin for structural study (31). In the end tissue, slides were studied to observe various pathological modifications such as inflammation, sinusoidal congestion, and pyknotic nuclei under a light microscope (magnification 10×) (MCX 100, Micros Austria).

### 2.8 Gene expression analysis

Gene expression was quantified in prepared cDNAs by performing quantitative real-time PCR (qRT-PCR) using the Maxima SYBR Green/ROX Master Mix on the iQ5 Bio-Rad system. Values obtained after PCR were analyzed *via* the 2*(–ΔΔct) method. Beta-Actin was used as a reference gene (Table 2).

**TABLE 2:** Primer sequences used in quantitative real-time for gene expression analysis.

Gene	Primer sequence
Beta-Actin F	CGAGTACAACCTTCTTGCAGC
Beta-Actin R	TATCGTCATCCATGGCGAACTG
MAPK-8 F	CTCAGCATCCGGTCTCTTCG
MAPK-8 R	CTGCTGTCTGTATCCGAGGC
Traf-4 F	CGACTACAAGTTCCTGGAGAAGC
Traf-4 R	AGGTGTCGCAGAAGCGGTG
Traf-6 F	GGCGCCTAGTAAGACAGGAC
Traf-6 R	GGCGCCTAGTAAGACAGGAC

### 2.9 Statistical analysis of data

Results obtained from different assays were statistically analyzed using software SPSS statistics 25 (32). Analysis of variance (ANOVA) under a completely randomized design was employed for *M. oleifera* and extract related parameters, whilst one-way ANOVA was used to check the level of significance followed by Tukey’s HSD (α: 5%) for the bio-evaluation trail.

## 3 Results

### 3.1 Phytochemical analysis of *Moringa oleifera*

**FIGURE 2:**
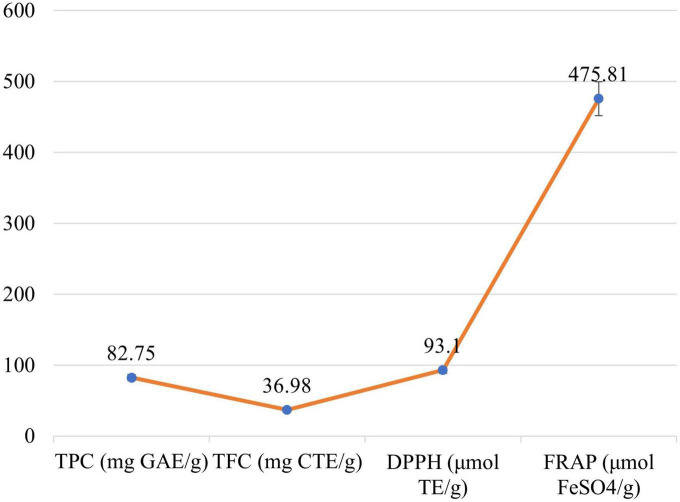
Antioxidant activity of *Moringa oleifera* leaves: TPC, TFC, DPPH, and FRAP.

Significant differences (*P* ≤ 0.05) were examined between the phytochemical content of *M. oleifera* leaves which is shown in Figure 2. Mean value for TPC in *M. oleifera* leave was calculated as 82.75 mg GAE/g. The TFC content of *M. oleifera* leaves was observed as 36.98 mg QE/g. To the inquire antioxidant potential of the *M. oleifera* leaves, 2,2-diphenylpicrylhydrazyl radical (DPPH•) scavenging and Ferric reducing the ability of plasma (FRAP) were performed. Leaves showed the DPPH• and FRAP as 93.1 μmol TE/g and 475.87 μmol FeSO_4_/g, respectively.

### 3.2 APAP administration induced acute liver injury in *Sprague Dawley* rats

A significant increase (*p* ≤ 0.05) in ALT, ALP, AST, bilirubin, and GGT levels was observed in the acetaminophen-treated group as compared to the control group as shown in Table 3. The rise in ALT, ALP, AST, bilirubin, and GGT value in hepatotoxic rats may be due to cellular damage in hepatic cells because of increased oxidative stress caused by APAP toxic dose. For serum proteins, albumin, globulin, and A/G ratio were observed. These results showed a significant change in serum globulin levels among all the groups (Table 4). The highest serum liver biomarkers level in APAP hepatotoxic rats is an indication of hepatic damage caused by the toxic metabolite of acetaminophen (33).

**TABLE 3:** Mean values for liver functioning tests.

Treatments	Liver weight (g/g body weight)	S.G.P.T. (IU/L)	Alkaline phosphatase (ALP) (IU/L)	Aspartate aminotransferase (AST) (U/L)	Serum bilirubin (mg/dl)	Gamma glutamyl transferase (GGT) (units/l)
G_0_	3.31 ± 0.22^B^	37 ± 3.62^B^	173 ± 7.54^B^	58.33 ± 3.05^C^	0.44 ± 0.01^B^	5.44 ± 0.21^C^
G_1_	3.89 ± 0.1^A^	61 ± 6.92^A^	218.33 ± 3.05^A^	111.67 ± 5.51^A^	0.65 ± 0.08^A^	7.07 ± 0.26^A^
G_2_	3.33 ± 0.19^B^	42.67 ± 3.51^B^	178.33 ± 8.73^B^	72.67 ± 5.85^B^	0.53 ± 0.01^B^	6.29 ± 0.15^B^
G_3_	3.15 ± 0.16^B^	41 ± 2.01^B^	176.67 ± 8.02^B^	64 ± 4.35^BC^	0.46 ± 0.01^B^	6.15 ± 0.25^B^
SEM	0.1024	2.5386	4.1533	2.7839	0.0243	0.1285

^A–C^The values carrying different letters within column are significantly different.

G_0_, normal control rats; G_1_, acetaminophen induced hepatotoxic rats; G_2_, silymarin pre-treated rats; G_3_, *Moringa oleifera* pre-treated rats.

**TABLE 4:** Mean values for serum proteins (albumin and globulin) and oxidative stress biomarkers (serum total oxidative stress and serum total antioxidant capacity.

Treatments	Serum albumin (g/dl)	Serum globulin (g/dl)	A: G	Serum total oxidative stress (μmol/L)	Serum total antioxidant capacity (mmol/L)
G_0_	4.63 ± 0.22	2.59 ± 0.17^B^	1.37 ± 0.05	17.84 ± 0.23^B^	2.09 ± 0.12^BC^
G_1_	3.57 ± 0.30	2.38 ± 0.25^B^	1.17 ± 0.05	22.94 ± 1.56^A^	1.50 ± 0.38^C^
G_2_	4.21 ± 0.23	2.75 ± 0.11^B^	1.47 ± 0.25	17.67 ± 1.26^B^	2.74 ± 0.23^AB^
G_3_	4.20 ± 0.08	3.37 ± 0.17^A^	1.27 ± 0.05	16.79 ± 0.39^B^	3.28 ± 0.28^A^
SEM	0.13	0.10	0.07	0.5966	0.1584

^A–C^The values carrying different letters within column are significantly different.

G_0_, normal control rats; G_1_, acetaminophen-induced hepatotoxic rats; G_2_, silymarin pre-treated rats; G_3_, *Moringa oleifera* pre-treated rats.

### 3.3 Hepatoprotective effect of *Moringa oleifera* on liver biomarkers

The pre-treatment of rats with moringa leaves significantly prevented the acetaminophen-induced increase in ALT, ALP and AST, bilirubin, and GGT levels consistent with the gross and histopathological changes in the liver. The decline of the enzyme values in the *M. oleifera* treated group may be a repairing effect of phytochemicals presented abundantly in the *M. oleifera* leaves. Acetaminophen abnormally elevated liver enzymes by disrupting normal cellular activity, which was protected by *M. oleifera* due to its antioxidant activity and its modulatory effect on free radicals. The moringa pretreated group showed significantly reduced (*p* ≤ 0.05) liver damage and normalized the architecture of the liver. Moringa also showed protection from APAP toxicity by significantly restoring the serum protein level (*p* ≤ 0.05) elevated by APAP. These results indicate that moringa pretreatment improved liver biomarker levels and conserved its activity (Tables 3, 4).

### 3.4 *Moringa oleifera* reduced APAP-induced hepatic injury by its antioxidant capacity

Knowing that APAP-associated hepatic injury involves oxidative stress, we wondered whether the protective effects of *M. oleifera* are due to its antioxidant potential. The statistical analysis showed that the serum total oxidative stress and serum TAC varied significantly in all the groups. The highest serum TAC was observed in *M. oleifera* pre-treated rats. The lowest antioxidant capacity was seen in rats with acetaminophen-induced hepatotoxicity. Low antioxidant activity is linked with increased oxidative stress, which further worsens the disease by activation of immune responses as a defensive mechanism. The highest antioxidant capacity of *M. oleifera* justified its therapeutic application as a hepatoprotective agent (Table 4).

### 3.5 Histopathological examination of liver

**FIGURE 3:**
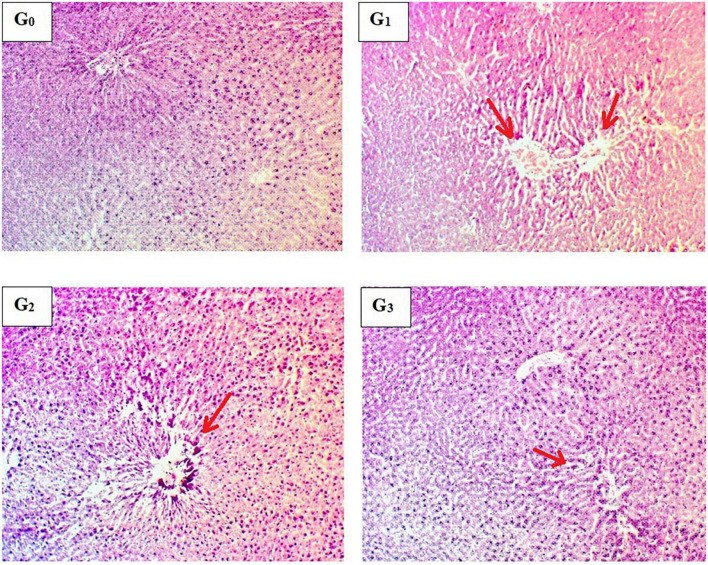
Histopathological indications of hepatic parenchyma. **(G_0_)** Normal control rats showing the normal liver structure, **(G_1_)** Acetaminophen-induced hepatotoxic rats showing vascular degeneration, **(G_2_)** Silymarin pretreated rats showing improvement in liver histology, **(G_3_)** *Moringa oleifera* pretreated rats showing the normal hepatocellular architecture.

Significant inflammatory and vascular changes along with sinusoidal modifications were observed during histopathological examination in APAP-induced hepatotoxic rats (Figure 3). Hepatic parenchyma appeared to be normal in normal diet-fed rats (G_0_). Normal sinusoidal spaces were observed in this group, where hepatocytes were properly arranged in hepatic cords. Furthermore, no pyknotic nuclei were identified. To determine the severity of liver damage by APAP exposure, a histopathological examination of the liver was carried out, which indicated abnormal morphological and degenerative changes after 8 h of APAP exposure. Severe cellular degeneration and necrotic changes were reported in APAP-induced hepatotoxic rats (G_1_). Gross examination of liver samples from Silymarin pretreatment (G_2_) exhibited slight modifications in liver structure following 8 h of APAP exposure. Likewise, the *M. oleifera* pre-treated group (G_3_) represented less vascular injury with normal hepatic parenchyma and less inflammation. The study concluded that pre-treatment with *M. oleifera* helps to attenuate hepatic degenerative changes in the severely damaged liver during APAP administration.

### 3.6 Gene expression analysis

#### 3.6.1 MAPK downstream JNK pathway

**FIGURE 4:**
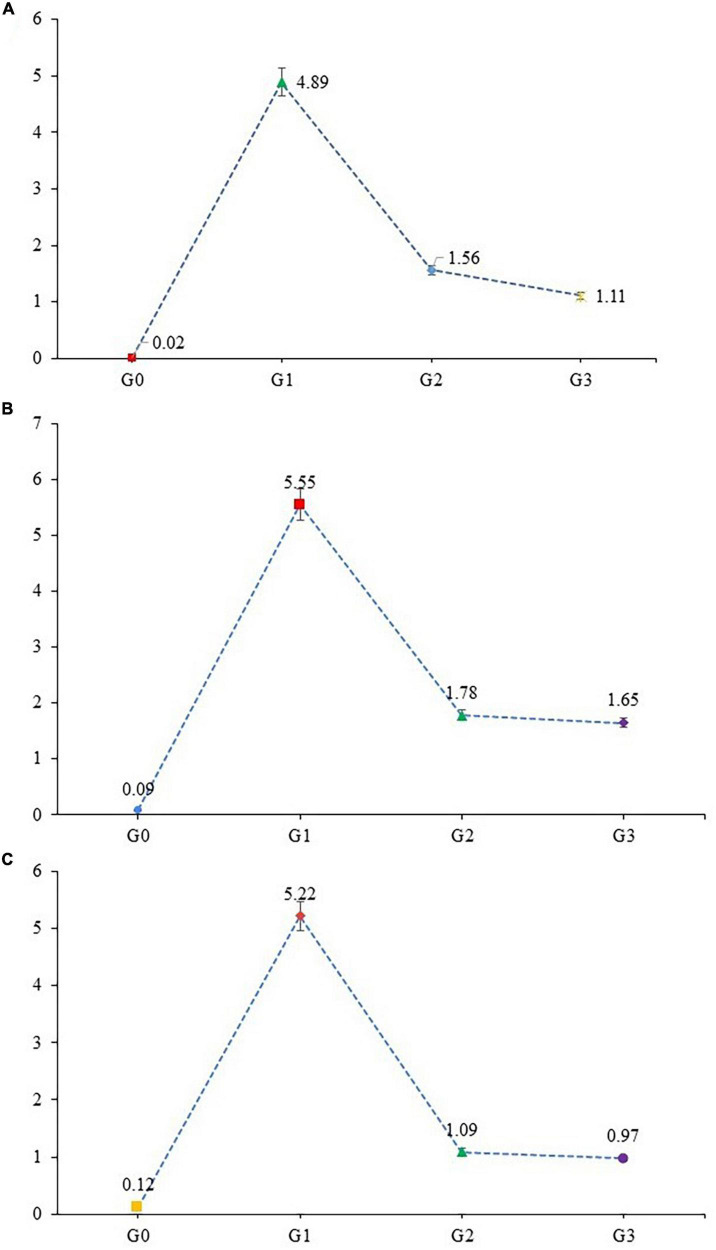
Gene expression of **(A)** MAPK-8, **(B)** TRAF-4, and **(C)** TRAF-6 gene (mean values) in liver tissues of experimental rats.

Stress-associated activation of MAPK along with initiation of the JNK pathway plays a major role in the adaptive and innate immune systems. Mitogen-activated protein kinases (MAPK) regulate the protein family, involved in cell proliferation, differentiation, and cell death. The expression level of MAPK-8, TRAF-4, and TRAF-6 were examined in liver tissue for MAPK downstream JNK pathway. The expression level of MAPK-8, TRAF-4 and TRAF-6 genes were significantly upregulated (Figure 4) in APAP hepatotoxic rats (G_1_) when compared with healthy rats (G_0_). In contrast the expression levels of MAPK-8, TRAF-4, and TRAF-6 genes were significantly down-regulated in G_2_ and G_3_, respectively. Over-expression of genes associated with MAPK downstream JNK Pathway, in APAP, treated group (G_0_) is the result of increased oxidative stress and cellular damage in organ, while a protective effect was observed in moringa-(G_3_) and silymarin-treated group (G_2_). Moringa in comparison to silymarin significantly downregulated the expression of MAPK-8 genes, while the effect of these two treatments was non-significant in TRAF-4 and TRAF-6 genes expression. Healthy rats showed minimal expression of MAPK downstream JNK pathway possibly due to the inactivation of MAPKs.

## 4 Discussion

Acetaminophen-induced hepatic injury is the most common cause of drug-induced liver diseases and acute liver failure in humans and experimental animals that is associated with oxidative stress and free radicals (34). Unfortunately, over-consumption of acetaminophen is associated with acute liver failure and drug-induced liver diseases in animal and human models (35). Saturation of metabolic pathways occurs at toxic doses that result in increased synthesis of toxic metabolite-acetyl-*p*-ben zoquinoneimine (NAPQI) and depleted natural antioxidant activity in the body (36). In this present study, biochemical characterization, and therapeutic action of the *M. oleifera* leaves, pods, and flower extract were investigated against APAP-induced hepatotoxicity in experimental rats.

There is vast heterogeneity of phenolic compounds in plant species. Raudone et al. (37) evaluated TPC content in 133 Indian medicinal plants in which they have found 1.8 mg GAE/g in *M. oleifera* seeds, which was quite lower than the present study (32.56 mg GAE/g). Another study conducted by Mwamatope et al. (38) showed similar results (12.23 mg GAE/g) of TPC in ethanolic extract of *M. oleifera* leaves. The variations in TPC values might be due to several factors such as different amounts of secondary metabolites, genotype, and plant maturity age. Bennour et al. (39) used different extraction techniques such as direct maceration (DM), successive maceration (SM), and different solvents i.e., dichloromethane, butanol, acetyl acetate, and aqueous on the phenolic composition of *M. oleifera* and found variations in TPC values of *M. oleifera* extracts. Yun et al. (40) conducted a study on phytochemical screening of *M. oleifera* leaf hydrolyzsate and they found the TFC values in the range between 17.23 and 27.83 mg QE/g. Pakade et al. (41) worked on the phytochemical potential of *M. oleifera* and declared that moringa leaves have the highest source of phytochemicals as compared to flowers.

The current study reveals that the extract of *M. oleifera* leaves exhibited radical scavenging activity to a certain level. The current findings are in line with the study conducted by Vyas et al. (42) who reported radical scavenging activity of 58, 83, and 39 μmol TE/g in flowers, leaves, and pods, respectively. APAP overdose results in depletion of glutathione and an increase in its metabolite (NAPQI) concentration (43). The characteristic markers of hepatotoxicity include elevated levels of AST, ALT, ALP, Bilirubin, and GGT (44). The augmented level of liver biomarkers is the key indication of liver injury. The current study concluded that pretreatment with moringa significantly restored these elevations due to its high antioxidant potential and its ability to scavenge free radicals (45). Similar outcomes were mentioned by Islam and Alam (46), where the protective activity of *M. oleifera* Lam. was evaluated in paracetamol administered hepatotoxic rats. A toxic dose (600 mg/kg BW) of APAP was orally administered to the positive control group once a week. The effectiveness of *M. oleifera* (250 mg/kg BW) was checked against the toxic APAP dose. The modulatory effect of *M. oleifera* extract was observed on S.G.P.T, ALP, S.G.O.T, and Bilirubin (33). An increase in plasma level of liver enzymes in the *M. oleifera*-treated group justifies the current research work, where *M. oleifera* has shown ameliorating effect on liver enzymes. Likewise, the study conducted by Sharifudin et al. (47) reported therapeutic benefits of *M. oleifera* against paracetamol-induced liver injury. Their findings were similar to this current research work as acetaminophen elevated liver biomarkers in serum, while *M. oleifera* potentially normalized them by their antioxidant nature. Mousa et al. (48) conducted a research trial in which the protective effect of *M. oleifera* ethanolic extract (MOLE) was evaluated. Phytochemical characterization revealed that MOLE is the richest source of phytochemicals such as quercetin, kaempferol, quercetin-3-*O*-glucoside, kaempferol -3-*O*-glucoside, kaempferol malonyl glucoside and ellagic acid (49). This antioxidant pool enables this plant to perform a physiological role in the body. In accordance with the current bilirubin results, previous studies indicated similar outcomes and concluded that *M. oleifera* is a preventive tool to regulate liver biomarkers including bilirubin (50). Similarly, results depicted that moringa and silymarin pre-treatment regulated serum proteins in APAP-administered rats, which indicates normal liver functioning in both treatment groups, as albumin and globulin both are produced by the liver and their normal production is a key marker for healthy liver functioning (51). Adding *M. oleifera* (200 mg/kg/BW) to the daily diet of experimental rats for 8 weeks significantly increased antioxidant enzymes concentration and normalized liver functioning enzymes (AST, ALP), serum total proteins, albumin, globulin, TAC) and total oxidative stress (TOS) (52).

Glutathione is the body’s major antioxidant enzyme produced naturally during oxidative stress. Toxic exposure to APAP significantly reduced glutathione concentration, ultimately enhancing total oxidative stress (TOS) (53). However, treatment with *M. oleifera* significantly enhanced TAC by enhancing glutathione production (54). An experimental assessment of *M. oleifera* leaf and flower was made on antioxidant, antistress, and scavenging potential. Aqueous extract of MO leaf potentially increased GSH and provided strong reducing power and free radical scavenging capacity, thus enhancing TAC. The imbalance of these free radicals imposes adverse effects on normal body tissues (55). For this purpose, histopathology is the key tool to inspect the extent and type of injury to an organ’s portfolio. Histopathological analysis of renal and hepatic tissues revealed that a toxic dose of APAP severely affected hepatic and renal parenchyma and resulted in condensed chromatids with enlarged hepatocytes. Furthermore, it may cause inflammatory infiltration and sinusoidal congestion, thus compromising liver and kidney functionality. Moreover, previous researchers highlighted the protective role of *M. oleifera* leaf against hepatic injury in APAP-exposed rats (26, 56) that triggered oxidative stress and modulated inflammatory cytokines in renal parenchyma. Several previous studies have confirmed that the administration of a toxic dose of acetaminophen is linked to an increase in ROS/RNS production and oxidative stress (33, 57, 58). Administration of APAP led to GSH depletion ultimately resulting in hepatic injury and oxidative stress in liver tissues (59). Following the cascade, the stress pathway i.e., MAPK downstream JNK pathway was activated by APAP exposure that can be assessed by increased expression of MAPK-8, TRAF-4, and TRAF-6. These findings are supported by the earlier findings of Saberi et al. (60) who reported upregulation of MAPK downstream JNK pathway genes in stressed conditions. Activation of this pathway is directly linked with an increase in intracellular oxidative stress, which in turn, proceeds the apoptotic process (61). It can be concluded from the above findings that acetaminophen-induced hepatotoxicity can be a possible cause of MAPK downstream JNK pathway activation. However, treating rats with *M. oleifera* significantly conserved the antioxidant pool by reducing the expression of MAPK downstream JNK pathway many folds, when compared with diseased rats.

## 5 Conclusion

In summary, we conclusively established that phytochemical enriched *M. oleifera* leaves effectively protects the liver from APAP by regulating natural antioxidant enzymes level to fight against the ROS generation. Furthermore, it normalized the elevated biochemical parameters such as liver functioning enzymes (ALT, AST, ALP), serum proteins, and oxidative stress biomarkers. Also, histology of hepatic parenchyma revealed that *M. oleifera* pre-treatment ameliorated cellular necrosis, and inflammatory changes and conserved normal hepatic structure. Pre-treatment with *M. oleifera* also improved liver performance by effectively inhibiting cellular stress signaling cascades and by downregulating the expression of MAPK-8, TRAF-4, and TRAF-6, which plays a vital role in the inflammatory process. These findings confirmed that *M. oleifera* leaves could be used as an effective antidote against acetaminophen-induced liver injury, as it suppresses key biomarkers involved in the hepatotoxicity pathway. However, further extensive research is still required to explore the underlying molecular mechanisms.

## Data availability statement

The original contributions presented in this study are included in the article/supplementary material, further inquiries can be directed to the corresponding author.

## Ethics statement

This animal study was reviewed and approved by Institutional Biosafety Committee (IBC) of University of Agriculture, Faisalabad, Pakistan Protocol Permission No: 1212/12-03-2021 National Biosafety Rules, 2005, Pakistan.

## Author contributions

NY: data curation and writing – original draft. MK: resources, supervision, and writing – review and editing. TZ and MF: writing – review and editing. All authors contributed to the article and approved the submitted version.
